# Sub-clinical detection of gut microbial biomarkers of obesity and type 2 diabetes

**DOI:** 10.1186/s13073-016-0271-6

**Published:** 2016-02-17

**Authors:** Moran Yassour, Mi Young Lim, Hyun Sun Yun, Timothy L. Tickle, Joohon Sung, Yun-Mi Song, Kayoung Lee, Eric A. Franzosa, Xochitl C. Morgan, Dirk Gevers, Eric S. Lander, Ramnik J. Xavier, Bruce W. Birren, GwangPyo Ko, Curtis Huttenhower

**Affiliations:** The Broad Institute, 415 Main St, Cambridge, MA 02142 USA; Center for Computational and Integrative Biology, Massachusetts General Hospital and Harvard Medical School, Boston, MA 02114 USA; School of Public Health, Seoul National University, 1 Gwanak-ro, Gwanak-gu, Seoul South Korea; Department of Biostatistics, Harvard School of Public Health, 655 Huntington Avenue, Boston, MA 02115 USA; The Broad Institute of MIT and Harvard, 415 Main St, Cambridge, MA 02142 USA; Samsung Medical Center, Sungkyunkwan School of Medicine, 25-2 Sungkyunkwan-ro, Jongno-gu, Seoul South Korea; Busan Paik Hospital, Inje College of Medicine, 197 Inje-ro, Gimhae-si, Gyeongsangnam-do, South Korea; Janssen Human Microbiome Institute, Janssen Research and Development, Cambridge, Massachusetts USA; Department of Biology, Massachusetts Institute of Technology, Cambridge, MA 02139 USA; Department of Systems Biology, Harvard Medical School, Boston, MA 02115 USA; Gastrointestinal Unit and Center for the Study of Inflammatory Bowel Disease, Massachusetts General Hospital and Harvard Medical School, Boston, MA 02114 USA; Center for Microbiome Informatics and Therapeutics, Massachusetts Institute of Technology, Cambridge, MA 02139 USA

## Abstract

**Background:**

Obesity and type 2 diabetes (T2D) are linked both with host genetics and with environmental factors, including dysbioses of the gut microbiota. However, it is unclear whether these microbial changes precede disease onset. Twin cohorts present a unique genetically-controlled opportunity to study the relationships between lifestyle factors and the microbiome. In particular, we hypothesized that family-independent changes in microbial composition and metabolic function during the sub-clinical state of T2D could be either causal or early biomarkers of progression.

**Methods:**

We collected fecal samples and clinical metadata from 20 monozygotic Korean twins at up to two time points, resulting in 36 stool shotgun metagenomes. While the participants were neither obese nor diabetic, they spanned the entire range of healthy to near-clinical values and thus enabled the study of microbial associations during sub-clinical disease while accounting for genetic background.

**Results:**

We found changes both in composition and in function of the sub-clinical gut microbiome, including a decrease in *Akkermansia muciniphila* suggesting a role prior to the onset of disease, and functional changes reflecting a response to oxidative stress comparable to that previously observed in chronic T2D and inflammatory bowel diseases. Finally, our unique study design allowed us to examine the strain similarity between twins, and we found that twins demonstrate strain-level differences in composition despite species-level similarities.

**Conclusions:**

These changes in the microbiome might be used for the early diagnosis of an inflamed gut and T2D prior to clinical onset of the disease and will help to advance toward microbial interventions.

**Electronic supplementary material:**

The online version of this article (doi:10.1186/s13073-016-0271-6) contains supplementary material, which is available to authorized users.

## Background

The human gut microbiota plays an important role in health and disease [1, 2] and can be viewed as a mirror into the host physiology. One of the primary roles of the microbiota is energy harvest; thus, it is not surprising that microbial dysbiosis has been associated with various metabolic disorders, including type 2 diabetes (T2D) [3, 4] and obesity [5–7]. T2D is often a consequence of obesity. As the diagnosis is threshold-based, risk of developing T2D in the near future correlates with high levels of two biomarkers, fasting blood sugar (FBS) and HbA1c, even when they do not meet the clinical criteria (HbA1c >6.5 % or FBS >125). However, the microbial changes that occur in the sub-clinical state, prior to the onset of disease, have never been examined, but may potentially be used for early diagnosis and intervention.

Previous profiles of the gut microbiome during clinical T2D have found compositional changes between patients and healthy controls [3, 4], including an obesity-related change in the abundance ratio of Bacteroidetes:Firmicutes [5, 8], and a decreased abundance of mucin-degrading *Akkermansia muciniphila* in overweight children [9] and pregnant women [10, 11]. However, there is no strong consensus across studies in taxa changing in obese versus lean individuals [12]. The causes for this inconsistency may be either technical or biological. From a technical standpoint, a lack of consistent standard operating procedures for sample preparation and sequencing can lead to great variance between different labs and studies [12]. Biologically, the specific composition of the community may be much less important than its overall functional capability.

Indeed, there is greater consensus between these studies when microbial functional dysbioses are considered rather than microbial composition [4]. The gut communities of T2D patients showed increased capacity for oxidative stress resistance, and a decreased capacity for flagellar assembly and riboflavin metabolism [3, 4]. Interestingly, oxidative stress resistance was also enriched in the guts of patients with inflammatory bowel diseases (IBD) [13], potentially indicating that the microbiome is generally stressed by low-level inflammation and immune activation, which may be present at the sub-clinical state of T2D as well.

Despite recent studies associating the microbiome with T2D [3, 4] and obesity [6, 8], all previous work has examined individuals with well-established disease. These data may be further influenced by additional factors, such as decreased subject mobility, and it is difficult to conclude from study design whether the observed microbial changes preceded the onset of disease. Furthermore, these studies have rarely taken into account the various genetic backgrounds of the patients. We have addressed these issues by performing the first metagenomic profile of the gut microbiome of monozygotic (MZ) twins, spanning the entire healthy range of T2D clinical indicators, including body mass index (BMI) and fasting blood sugar (FBS). Identifying gradient-like associations between these parameters and gut microbiome features in the sub-clinical state of these diseases will open the way to discover potential markers for early diagnosis of T2D and obesity.

We found several taxa associated with sub-clinical changes in BMI, blood pressure, sugar, and triglycerides, including enrichment of the *Roseburia* genus and depletion of the *Akkermansia muciniphila* species. Additionally, riboflavin and NAD biosynthesis were metagenomically enriched in participants with high blood pressure and BMI values. Interestingly, similar functional enrichments are shared with other gut inflammatory conditions such as IBD [13] and clinically-established T2D [3], suggesting shifts in the gut microbial population prior to full disease onset that may be either causal or an early correlative indicator.

Finally, this cohort included a unique combination of MZ twins and longitudinal sampling, which allowed us to identify the degree to which specific microbial strains were shared between the guts of siblings and maintained over time. Despite the small size of this targeted cohort, the deep metagenomic sequencing (mean 3.5 Gnt per sample) combined with a focus on taxa of high relative abundance (see Methods) enabled us to determine strain similarity with high resolution in these data. MZ twins have also been previously observed to share a greater proportion of gut microbes than unrelated individuals [6], and some strains appear to be maintained within the guts of isolated individuals for months to years [14]. Surprisingly, we observed that while twins in our MZ cohort indeed share a substantial subset of microbial species, strains within these species differ between related twins. Thus, the gut microbial similarities of twins may arise from sources such as genetic pressure to acquire certain species (but not specific strains), or from early colonization by the same strains, with subsequent genetic divergence over the course of a lifetime.

### Ethical consent

Written informed consent was obtained from each participant. The study protocol was approved by the institutional review board (IRB) of Samsung Medical Center, Busan Paik Hospital, and Seoul National University (IRB No. 144-2011-07-11).

## Methods

### Study participants and specimen collection

The participants were MZ twins who enrolled for the Healthy Twin study in Seoul and Busan, South Korea. The zygosity of twins was determined using AmpFlSTR Identifier Kit with 16 short tandem repeat markers (15 autosomal STR markers + one sex determining marker) or a questionnaire with a validated accuracy of >90 % [15]. Details on methodology of this cohort have been previously described [16].

A total of 36 fecal samples from the participants were collected: samples from two twin pairs were taken once and those from eight twin pairs were obtained twice with an average interval of 2 years. Twins were in the age range of 30-48 years at the first sampling point. Fecal samples were taken in conjunction with a health examination and immediately stored at -25 °C. They were subsequently transported to the two central clinics and stored at -80 °C until DNA extraction. Blood samples were drawn by vein puncture after an overnight fast and sent to a central laboratory to measure biochemical factors.

During each visit, individuals also completed a questionnaire recording life style, medication, and dietary habits. Anthropometrical measurements (height, weight, waist circumference, and so on) and biochemical tests (glucose, hsCRP, total cholesterol, HDL-C, LDL-C, triglyceride, and so on) were also conducted (Additional file 1: Table S1). The derived homeostasis model assessment (HOMA) index uses the fasting blood sugar and insulin to predict the insulin resistance of patients [17] and was calculated as standard (insulin * glucose)/405, both measured after fasting and glucose levels measured in mg/dL [17].

### Nucleic acid extraction and metagenomic shotgun sequencing

Total DNA was extracted from each fecal sample using the MoBio Power Soil DNA Isolation kit (MoBio, Solana Beach, CA, USA) according to the manufacturer’s instructions and stored at -80 °C until subsequent analysis. All samples were sequenced using the Illumina Hiseq2000 instrument, which produced paired-end reads of 101 nt, yielding average 3.5 Gnt per each of fecal samples.

### Metagenomic shotgun sequences analysis

The gut microbial composition of each sample was profiled using MetaPhlAn [18]. MetaPhlAn uses a unique set of markers for each species (and higher level clades) to estimate the abundance of species in each sample according to the number of mapped reads to its markers. The relative abundances of the gut microbial functional pathways from metagenomically sequenced communities were determined using HUMAnN [19]. HUMAnN maps the sequenced reads to a non-redundant set of genes extracted from the KEGG database [20] and estimates the abundance level of each functional module by the number of matches to member genes fully compatible with it being carried out by one or more microbes.

### Testing for significant associations with the clinical metadata variables

To identify significant associations between microbial and phenotypic variables, we applied a linear multivariate regression model specifically adapted to microbiome data (MaAsLin, Multivariate microbial Association by Linear models [13]). MaAsLin constructs boosted, additive general linear models to associate metadata and transformed microbial taxonomic or functional relative abundances. Since microbial community profiles are typically high-dimensional, boosting is used for feature selection over potential covariates to identify those most associated with each microbial feature. Selected metadata are then used in a general linear model with metadata as predictors and arcsin-square root transformed microbial relative abundances as the responses. In this study, model covariates of interest comprised of clinical variables included in Additional file 1: Table S1, and each model also included age, smoking status, sex, and twin as potential confounders (the latter as a random effect to accommodate repeated longitudinal measures).

### Comparing strains between samples

We performed taxonomic profiling with MetaPhlAn [18]. Briefly, MetaPhlAn operates by mapping raw sequence reads to a database of pre-defined clade-specific marker genes. Markers are those genes occurring in isolates from a particular clade but not outside of that clade. After mapping reads to clade-specific marker genes, the resulting raw counts are normalized for total marker gene length and outliers, yielding profiles of: (1) clade relative abundance; (2) marker gene presence/absence; and (3) marker gene abundance (in reads-per-kb (RPK) units, where 10 RPK would correspond to about ×1 coverage, given our 100 bp reads). Due to gene gain and loss events, an individual strain will not necessarily carry all of the markers associated with its corresponding species. A specific pattern of marker presence and absence can therefore be used as a molecular ‘barcode’ to identify a strain across samples. We next compared the marker gene abundance profiles of various samples (unrelated, twin or self; with median marker abundance >5 RPK) using a Bray-Curtis distance.

### Generating and analyzing the taxon-function correlation matrix

Spearman correlation was calculated between the profiles of each microbe and each function to generate the taxon-function correlation matrix. The KEGG database [20] was used to identify the ‘encoded’ correlations, by calculating the fraction of its reference sequences that include sufficient genes for any given module. The microbial co-occurrence matrix was calculated using spearman correlation between all taxa profiles to identify the ‘associated’ correlations.

### Comparing Korean and Western microbial populations

The prevalence and average abundances of all clades were calculated within our cohort and the HMP [21], and these were compared using Pearson correlation. Prevalence was defined as percent of samples with >0.001 relative abundance for each species, and average abundance was calculated only for samples passing that criterion.

### Sequence accession numbers and availability

Sequences generated in this study are publicly available at the European nucleotide archive (ERP002391).

## Results

### Monozygotic twin cohort and longitudinal metagenomic profiles

**Fig. 1:**
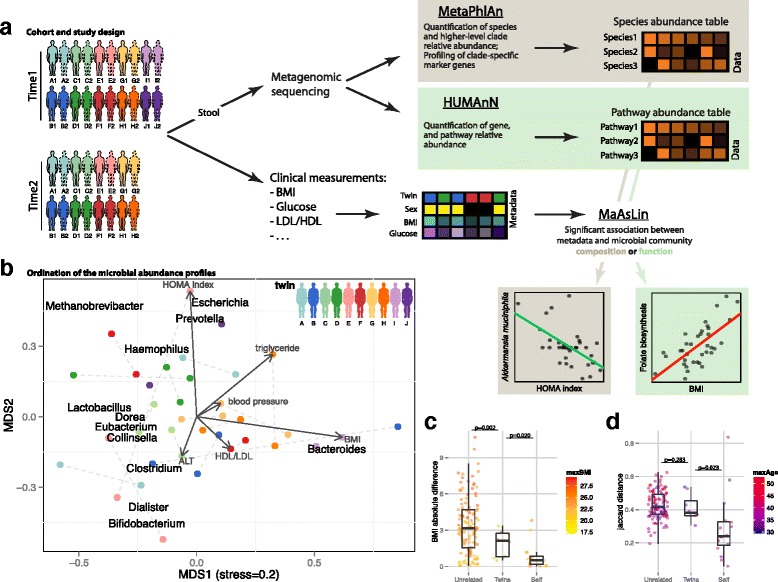
Study design for sub-clinical gut microbiome analysis in obesity and type 2 diabetes. **a** Stool and blood samples were collected at one to two time points from 10 MZ twin pairs. DNA was extracted from the stool samples and used for shotgun metagenomic sequencing, from which community composition and function were profiled using MetaPhlAn [18] and HUMAnN [19], respectively. Clinical biomarkers including sugar metabolism measurements (fasting blood sugar (FBS) and insulin (FBI)), inflammation markers (hsCRP) and others (Additional file 1: Table S1) were derived from accompanying blood samples. Finally, we determined significant associations between these clinical biomarkers and microbial taxa and functions using MaAsLin [13]. **b** Overall covariation of taxonomic profiles and the clinical biomarkers and taxa enriched among distinct sample subsets. Points represent samples ordinated using metric multidimensional scaling (MDS) by Bray-Curtis dissimilarity, colored by twin pair, with lines connecting samples from the same individual at different time points. Taxa and metadata are labeled at the point of maximum enrichment among samples. **c** Absolute BMI differences between any two ‘Unrelated’ (at time point 1), ‘Twins’ (at time point 1), and the same individuals at the two different time points (‘Self’). Comparisons are colored by the maximal BMI of the participants involved; *P* values were calculated using a *t*-test. **d** Taxonomic profile similarities of unrelated, twins, and individuals over time. Comparisons are colored by the maximal age of the participants involved; *P* values were calculated using a *t*-test

We collected fecal samples from 20 MZ Korean twins (10 twin pairs) at up to two time points each (12-44 months apart), resulting in 36 samples sequenced using metagenomic shotgun sequencing. In addition, multiple clinical parameters were measured at each sampling point, including body mass index (BMI), fasting blood sugar (FBS), cholesterol levels (LDL, HDL), fasting blood insulin (FBI), and renal and liver function (Additional file 1: Table S1), spanning the typical healthy range of these variables (Additional file 2: Figure S1). Species-level microbial abundance profiles were inferred using MetaPhlAn [18], and functional gene and pathway abundance profiles were generated using HUMAnN [19]. These microbial and functional profiles were tested for statistically significant association with clinical parameters using the MaAsLin [13] sparse multivariate linear model (Fig. 1a).

As is typical in the gut, the taxa that vary most widely in our cohort were members of the Bacteroidetes, Firmicutes, and at lower abundances the *Esherichia*, *Methanobrevibacter*, and *Bifidobacterium* genera (Fig. 1b). The x-axis depicts mostly the Firmicutes (left) to Bacteroidetes (ratio), as observed before [22], with a modest positive association between *Bacteroides* and BMI (see below). The y-axis is dominated by less prevalent or more variable genera, like *Methanobrevibacter* (25 % prevalence) and *Bifidobacterium* (2.7-3.3 coefficient of variation; Additional file 3: Table S2). The *Esherichia* and *Prevotella* genera are prevalent in our cohort (92 and 81 %, respectively, see Additional file 3: Table S2), but only a few individuals have high abundances of one or both genera, driving their contribution to population variability. Overall, we found that the prevalence and abundance profiles of the various taxa in our data are consistent with those measured in Western populations [21]; *P* value = 0.0001 by Pearson correlation; Additional file 4: Figure S2).

In our cohort, clinical parameters such as BMI (Fig. 1c) and microbial community composition (Fig. 1d) were both more similar between twins than between unrelated individuals, and both were self-similar over time. BMI is most stable between ‘self’ samples, especially since no individual has become obese during this study, and indeed, twins are more concordant on BMI compared to unrelated [6]. When comparing their microbial composition we found that, as expected, twins were somewhat more similar than unrelated [6], but self-samples were significantly more stable [21], indicating that for both clinical and microbial phenotypes, longitudinal samples were more similar than twins, which were in turn more similar than unrelated.

### Phylogenetic and functional diversity in the Korean gut microbiome

**Fig. 2:**
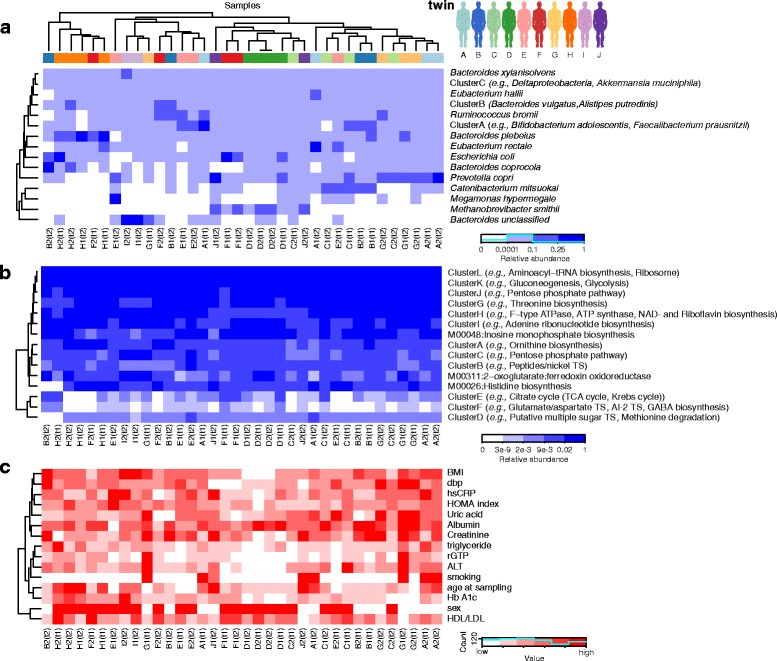
Taxonomic and functional profiles of twin gut microbiomes accompanied by T2D/obesity clinical indicators. **a** Clustered taxonomic profiles, discretized and with groups of tightly covarying taxa binned into 15 clusters for visualization. Each row represents a cluster of one or more species, and each column is one sample colored by the twin variable. Values indicate relative abundances from the medoid member of each cluster (see Additional file 5: Figure S3 for full matrix). **b** As (a) for metabolic modules derived from metagenomic functional profiling. Sample clustering retains ordering from (a). **c** Corresponding clustering of selected discretized clinical biomarkers, again retaining ordering from (a)

Several organisms were prevalent (present in >50 % of individuals) in this cohort, although often at relatively low abundance levels. Some of these are shared with other globally surveyed populations, while others were unusually prevalent in this population (Fig. 2a). Shared organisms included *Eubacterium rectale*, *Roseburia intestinalis*, and *Faecalibacterium prausnitzii*, which are similarly prevalent in our cohort and in Western population (94-96 %, 83-89 %, and 96-97 %, respectively) and with similar relative abundances (5 %, approximately 1 %, and 2-4 %, respectively), confirming the similarity between this cohort and Western population.

Less prevalent organisms included *Methanobrevibacter smithii*, the predominant archaeon in the human gut [23] with the important role of reducing the hydrogen via methanogenesis. *M. smithii* here mirrored its relatively low prevalence as seen in Western populations (25 %, compared to 21 % in the Human Microbiome Project (HMP)) [21], but with higher average relative abundance when present (9 %, compared to 0.3 %) [21]. Despite the major differences in the Korean diet and environment, *M. smithii* is still the major microbe likely responsible for this function.

Several organisms, including *Bifidobacterium longum*, *Escherichia coli*, *Prevotella copri*, and *Bacteroides plebeius*, were significantly more prevalent in our population compared to that of the HMP (Additional file 3: Table S2). It has been previously shown that *Prevotella* is more common in international cohorts [24], and indeed we found *P. copri* present in 81 % of our samples, compared to 16 % in the HMP [21]. Moreover, we found lower abundance of *P. copri* in our cohort when present (approximately 10 % relative abundance), unlike the high 37 % relative abundance found in the individuals who carry it in the HMP’s Western population [21], although the overall average of *P. copri* relative abundance in all individuals is similar between cohorts (6-8 %).

Another difference between the Korean and Western populations was *Bifidobacterium longum*, a microbe that ferments sugars into lactic acid noted as one of the first colonizers of the infant gut [25]. It was carried at an unusually high prevalence of 94 % (with average relative abundance of 2.5 %), in contrast to its presence in the HMP of 59 % prevalence and 0.4 % relative abundance [21]. *Bacteroides plebeius* was likewise enriched here, with 97 % prevalence in our cohort and only 9 % in the HMP [21]. *P. plebius* has been previously found in Japanese populations, likely due to its capability to break down complex carbohydrates specific to seaweed [26], and it may play a similar role in the guts of Koreans, as this is a major staple of their diet.

As has been previously observed [21], despite variability in the composition of the microbiome among these individuals, the distribution of microbial metabolic processes remained relatively stable (Fig. 2b, Additional file 5: Figure S3). One of the most variable modules was the transport system of autoinducer-2 (AI-2), a quorum-sensing signaling molecule traditionally associated with the Enterobacteriaceae and Vibrionaceae [27] and recently characterized in some *Bifidobacterium* species [28]. We also see striking variability in the biosynthesis of GABA, a major neurotransmitter in the central nervous system that has also been recently shown to be produced by some *Bifidobacterium* species [29]. Both these modules had a striking 3.8 coefficient of variance and approximately 18 % prevalence (compared to 94 % and 60 % in Western population, respectively) and shared similar abundance profiles in our data, suggesting a potential link between the two processes, perhaps through carriage by specific *Bifidobacterium* strains. As a control, we also see several ubiquitous ‘housekeeping’ processes such as the ribosome and translation, glycolysis, and gluconeogenesis were present at high levels with low variability among individuals (>4 % average relative abundance and 100 % prevalence, Additional file 3: Table S2 and Additional file 5: Figure S3).

### Host factors such as BMI associate with some microbes and processes in a graded fashion

**Fig. 3:**
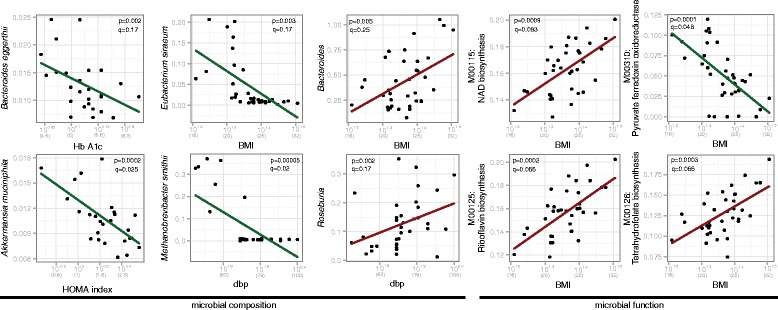
Selected significant associations of clinical markers with clade and pathway abundances. Lines represent linear model fit after transform to accommodate compositional, non-normally distributed data (see Methods) and account for age, sex, smoking, and twin relationships as covariates. Nominal *P* values and FDR corrected *q*-values are assigned by MaAsLin [13]. See Additional file 5: Figure S3 for complete list of significant associations

We investigated the relationship between host clinical phenotype and the gut microbiome by identifying significant multivariate linear associations using MaAsLin [13] (Fig. 3, Additional file 6: Table S3). This model associates microbial clade or pathway abundances with metadata of interest (for example, BMI, FBS, triglyceride) while accounting for other covariates (in this case sex, smoking, age, and the twin pairing; see Methods and Additional file 7: Figure S4). The abundance of *Akkermansia muciniphila* was negatively correlated with BMI, FBS, and insulin levels, for example, all in gradients ranging continuously over the ranges of these clinical variables and its relative abundance. This mucin-degrading microbe has been observed to be reduced in the guts of obese mice [30], pregnant women [10, 11], and overweight children [9], but this is the first time this trend has been observed in non-pregnant adults, especially within the normal range of BMI and FBS. This suggests the organism may represent one aspect of the obese gut microbiome that may be of specifically sub-clinical significance.

Other continuous associations of clades with phenotypes included a positive correlation between the *Bacteroides* genus and BMI, again spanning a range of the latter outside of clinical obesity. The Bacteroidetes:Firmicutes ratio is one of the earliest features of the gut microbiome suggested to associate with obesity in mice [31], but in this and other studies [11, 32, 33] associating an increase in Bacteroidetes with obesity rather than an increase in Firmicutes. Both positive and negative associations with the Bacteroidetes have been found in human populations [34], suggesting that this finding is not generalizable and depends greatly on factors that may include the underlying demographics, diet, sample preparation, and analysis (see Discussion).

We found multiple associations between microbial molecular function and clinical phenotypes (Fig. 3, Additional file 6: Table S3), including an increase in riboflavin-, NAD-, and tetrahydrofolate-biosynthesis and a decrease in pyruvate ferredoxin oxidoreductase accompanying increasing BMI levels (Fig. 3). Riboflavin and NAD are both required for the biosynthesis of the reduced form of glutathione (GSH) [35], an important antioxidant that alleviates the damage done by reactive oxygen species, and indeed we found that glutathione biosynthesis also increased at higher BMI levels (Additional file 6: Table S3). The direction of these associations suggests that the gut microbiota is producing more glutathione, and potentially processing more from the host, to relieve the increased oxidative stress at high levels of BMI.

Besides the gradient associations discussed above, we also identified two unusual threshold-like associations: BMI with *Eubacterium siraeum* and blood pressure with *Methanobrevibacter smithii*. In both these cases, the microbe is present only below a certain threshold (22.5 BMI, and 70 diastolic blood pressure, respectively). Interestingly, these trends do not appear in data collected for the human microbiome project (Additional file 8: Figure S5) and maybe unique to our cohort. Such discrete associations can be very interesting to investigate further, to potentially reveal the mechanism underlying the microbe’s sensing of host conditions.

### Contribution of specific microbes to overall gut community function

**Fig. 4:**
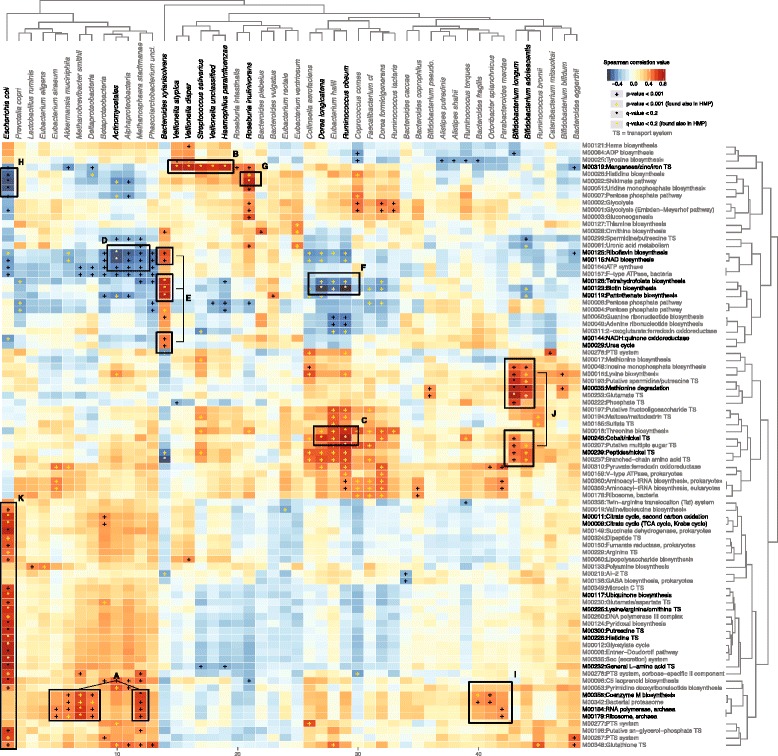
Association of taxa with microbial metabolic modules. The relative abundances of 56 total species were Spearman correlated against those of 87 functional profiles to identify covariation between taxa and metabolic modules (either due to genetic carriage or shared environment). Pluses and stars indicate nominal *P* value <0.01 or FDR *q*-value <0.2, respectively. Yellow marks indicate correlations also found in the corresponding analysis of HMP data [21] (see Additional file 11: Figure S8). Highlighted boxes are discussed in the main text

To better understand the relation between the taxonomic compositional profiles and function, we correlated the profiles of each clade with the profiles of each module (Fig. 4, selected individual scatter plots appear in Additional file 9: Figure S6). This allows us to hypothesize which microbes contribute to, depend on, or associate with specific metabolic and biomolecular processes carried out by the gut microbiota. In particular, a positive correlation between a module and microbe can have two explanations. In some cases, the function may be encoded in the microbe’s genome (referred to as ‘directly encoded’ correlations, Additional file 10: Figure S7A). Alternatively, a microbe might correlate with a function not because it carries it itself, but because it associates with other microbes that encode it (‘indirectly associated’ correlations). The former indicates microbes that perform a particular molecular process, the latter those that depend on its presence elsewhere in the community.

As expected, many ‘encoded’ correlations are found in our data and each of them induces others that are ‘associated’, based on the species co-occurrence network as computed using Spearman correlation (Additional file 10: Figure S7B). One interesting ‘encoded’ example is a set of archaeal functions, such as coenzyme M biosynthesis and archaeal RNA polymerase and ribosome, correlated with the archaeal species *Methanobrevibacter smithii* and *Methanosphaera stadtmanae* (Fig. 4 Box A). Correspondingly, we found ‘associated’ correlations between these archaeal functions and the abundance of Deltaproteobacteria, *Akkermansia muciniphila* and *Eubacterium siraeum*, which co-occur with the archaeal species in our data (Fig. 4 Box A; Additional file 10: Figure S7B Box A). We do not yet know why particularly these microbes would tend to share the archaeal environment, but it is interesting to examine if this relationship holds in other cohorts as well.

Additionally, three metal transport systems (TSs) were correlated with specific taxa. The manganese/zinc/iron TS M00319 is an ABC transporter, comprising four proteins, found originally in *Treponema pallidum* [36], and this specific TS is encoded only in the *Veillonella* species in our data. In addition to ‘encoded’ correlations between this TS and three *Veillonella* species, we identified two ‘associated’ correlations with *Streptococcus salivarius* and *Haemophilus parainfluenzae*, co-occurring species with the *Veillonella* (Fig. 4 Box B; Additional file 10: Figure S7B Box B). Similarly, the cobalt/nickel TS M00245 is another four-protein ABC transporter, estimated to be the most widespread uptake system for the two metals [37]. This module is encoded in many microbial species, and specifically in *Eubacterium hallii* and *Ruminococcus obeum* [38] in our communities, resulting in those encoded correlations. These were accompanied by additional ‘associated’ correlations with co-occurring species including *Dorea longicatena* (Fig. 4 Box C; Additional file 10: Figure S7B Box C). Lastly, two modules involving nickel TSs (cobalt/nickel M00245 and peptide/nickel M00239), together with sugar and amino acid metabolism and TS modules, were ‘encoded’ correlations found in the *Bifidobacterium* species. Methionine degradation M00035 was also encoded by these organisms, which generates *S*-adenosyl-L-methionine (SAM), a major methyl donor in the cell [39], and *Bifidobacterium* is used outside of the gut as a source of SAM in the functional food industry [40].

This cohort’s microbial co-occurrence network (Additional file 10: Figure S7B) can also explain some of the negative associations found in our data. For example, the abundance of *Bacteroides xylanisolvens* is negatively correlated with several taxa, including *Methanosphaera stadtmanae* and *Ruminococcus obeum* (Additional file 10: Figure S7B Box D), resulting in a negative correlation between these species and modules encoded by *B. xylanisolvens*, like NAD-, tetrahydrofolate-, and biotin biosynthesis (Fig. 4 Boxes D, E, F). Another example is the negative correlation between *Roseburia intestinalis* and *E. coli* (Additional file 10: Figure S7B Box E), resulting in the negative associations between *E. coli* and the Shikimate pathway, encoded in *R. intestinalis* genome sequence (Fig. 4 Boxes G, H). Such negative associations can also be the result of conflicting functionalities between certain microbes and metabolic functions not present in their genomes.

Interestingly, some correlations are neither ‘encoded’ nor ‘associated’, and we can only hypothesize as to their cause. One such example is the correlation between the archaeal functions mentioned above and abundance of *Bacteroides fragilis, Odoribacter splanchnicus* and *Parabacteroides merdae* (Fig. 4 Box I). Although these functions are not encoded in any of these genomes, the gene comB (2-phosphosulfolactate phosphatase), which belongs to the coenzyme M biosynthesis module, is encoded in the genome of *Parabacteroides merdae* [38], potentially explaining this association. Such a correlation might arise due to these organisms’ metabolic dependence on a function encoded by diverse organisms in different hosts.

Another interesting example is the positive correlation between riboflavin biosynthesis and the abundance of *Bacteroides xylanisolvens* (Fig. 4 Box E). As discussed above, we found several compositional and functional associations with BMI, including *Bacteroides*, riboflavin-, NAD-, and tetrahydrofolate- biosynthesis. Indeed, the latter two are ‘encoded’ correlations with *B. xylanisolvens*, suggesting that the increase in this species abundance in higher levels of BMI is contributing to the increase in NAD- and tetrahydrofolate- biosynthesis. Additionally, riboflavin biosynthesis may also be an ‘encoded’ association of *B. xylanisolvens* and we failed to identify it as such, potentially due to the incompleteness of current functional databases.

Finally, additional ‘encoded’ correlations were found between various amino acid transport systems and *E. coli* (Fig. 4 Box K). Many of these were detected in at least some strains of *E. coli*, due to the combination of *E. coli*’s very large pan-genome and the extent to which its strain variation space is well-covered by the many available reference genomes. Many of these correlations, like all of this cohort’s microbe-function correlations, were also found in the HMP [21] (yellow marks in Fig. 4, and Additional file 11: Figure S8). This suggests both that simple ‘encoded’ correlations recur across populations, as expected, and that more subtle ‘associated’ microbial dependencies may be consistent among diverse gut ecologies.

### Microbial species, but typically not strains, are shared between twins

Several studies have observed that related individuals, and particularly twins, carry more similar microbial communities than do unrelated individuals [6], and we reproduce this finding in our cohort (Fig. 1d). However, it has not been previously determined whether this similarity is due to ecological pressures that select for similar microbes among individuals, dispersal effects that cause the acquisition of identical microbes, or other factors. In cases where twins in this study shared similar taxonomic profiles and identical species, we thus tested whether these species were of the same strain. Defining microbial clades at genus-, species-, or strain- level is a difficult task [11, 41], and here we chose to define a strain as a combination of genomic markers, allowing us to identify dominant, near-clonal populations. Microbes were strain-typed within samples by identifying conserved or differential patterns of unique mobile element loss and gain using MetaPhlAn (see Methods), which has previously been successful in differentiating strains among individuals and over time [21, 42]. In particular, individuals were shown in these previous analyses to often carry a single dominant strain of most species [42] and for that strain to be significantly stable over time [14, 21] (Additional file 12: Figure S9). Applying this method to our data allowed us to determine: (1) when twins shared the same strain, in addition to the same species; and (2) when these strains were retained within an individual over time.

**Fig. 5:**
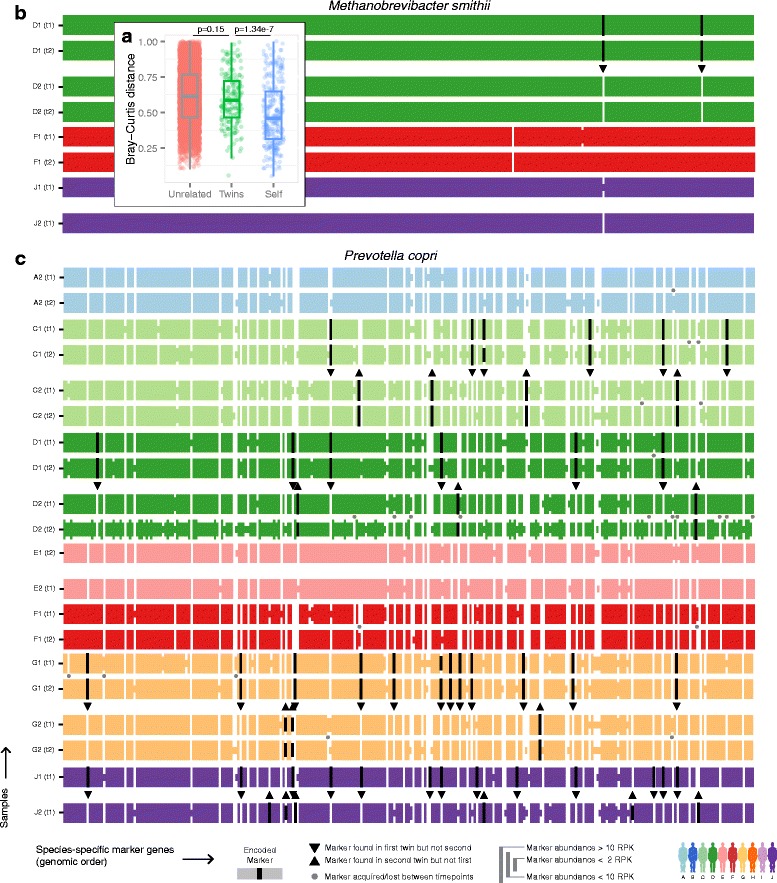
Strain profiles between unrelated, twins, and samples from the same individual over time. **a** Bray-Curtis dissimilarities of marker gene profiles indicative of strain similarity between unrelated, twins, and the same person over time (*P* values comparing unrelated vs. twins and twins vs. self are calculated by *t*-test). **b, c** The abundances of (**b**) *Methanobrevibacter smithii* and (**c**) *Prevotella copri* marker gene [18] profiles (see Methods). Each row represents one sample, colored by twin, containing vertical lines each representing one species-specific marker gene’s abundance as binned into four levels. Markers that are present only in one of the twins appear in black, with a triangle pointing to the absent (white) marker. Markers that differ between samples of the same person over time are marked with a gray dot

Remarkably, twins were not significantly more similar than unrelated in their strain composition (*P* = 0.15 by *t*-test), although (as expected) samples from the same individual over time were significantly similar (*P* = 1.34e-7; Fig. 5a). This suggests that either there is a genetic tendency for twins to retain broadly similar microbial compositions - but that this does not extend exert selective pressure at the strain level - or that identical strains acquired earlier in life during colonization have, by adulthood, evolved sufficiently to differ at multiple genomic elements. Only in rare cases did strains differ within individuals over time (Additional file 13: Figure S10), concordant with occasional sweeps of a replacement strain due to, for example, gene acquisition/loss or transfer from an external source.

We quantified the degree to which each microbial species was represented by the same or different strains among twins, specifically by calculating for each microbe the mean distance across genomic elements of all sample pair-wise comparisons (Additional file 14: Figure S11, see Methods). While some species retained very similar strains among twins (for example, *Methanobrevibacter smithii*, Fig. 5b), others consistently had more distinct strains (for example, *Prevotella copri*, Fig. 5c). While this detailed analysis is only possible in microbes that are particularly abundant in multiple samples, it raises the intriguing possibility that identical strains acquired early in life persist in the gut, but evolve rapidly through the gain and loss of genetic elements.

## Discussion

Here, we analyzed metagenomes from 36 fecal samples drawn from healthy Korean MZ twins over time, identifying associations between T2D-related biomarkers (for example, BMI, FBS) and microbial clades and functions. We found, among other examples, that BMI was negatively correlated with the abundance of *Akkermansia muciniphila* and positively correlated with riboflavin and NAD biosynthesis. These associations occurred over both the pre- and post-onset range of T2D clinical markers, suggesting that the microbiota may contribute to or react to changes in the host environment prior to the onset of disease. Furthermore, functional changes in the gut microbiome at higher sub-clinical values of BMI, FBS, and triglycerides resembled the signatures found in patients with established IBD or T2D, suggesting a shared response to oxidative stress in the gut, induced even at low levels of inflammation or immune activation. Finally, we found that while twins were more similar than unrelated individuals in microbial composition, they often carried different strains of these species. The computational framework presented here can be easily applied to other MZ twin cohorts, identifying early microbial markers of various other diseases, even in their sub-clinical phase.

Obesity and metabolic syndrome have long been associated with chronic, low-grade inflammation [43]. For example, macrophages of obese individuals accumulate in adipose tissue, where they express pro-inflammatory cytokines such as TNFa, IL6, and INOS [44], and the gut microbiota can initiate the inflammation and insulin resistance associated with obesity [34]. Interestingly, although there was no indication of host inflammation in our data (as measured by hsCRP), we observed the microbiome responding to this in the form of decreased abundance of *Akkermansia muciniphila* and increased NAD- and riboflavin-biosynthesis. This collection of functional changes together specifically enables the recharging of glutathione to its reduced form, promoting redox homeostasis in microbes potentially exposed to an increasingly hostile, inflammatory, oxidatively stressed environment the gut.

It is likely that additional compositional and functional shifts accompany this low-grade inflammation in T2D and related conditions, which will be better detected in other, larger cohorts capturing an even broader range of phenotypes and disease states. Finding common microbial changes is an important step towards understanding the cross talk between the gut microbiota and the diseased host, but whether these shifts are causal, responsive, or both remains an open question. For example, the microbial response to redox stress is more likely to be reactive, but microbes that are robust to this environment may promote its maintenance and thus contribute to immune activation or obesity. A combination of interventions in model systems and longitudinal prospective cohort studies of high-risk individuals, identifying the microbial changes that occur before the onset and during the early progression of the diseases, will enable us to determine whether the microbial shifts trigger host symptoms, or vice versa, and potentially by what specific molecular mechanisms.

**Fig. 6:**
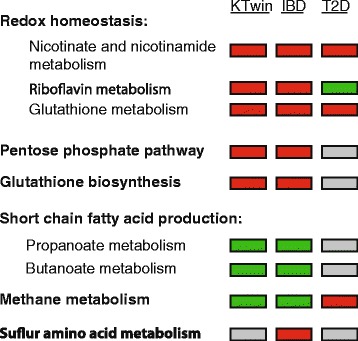
Microbial functional dysbioses common among this study and the gut microbiome in IBD and T2D. Several microbial metabolic pathways were determined to be significantly enriched (*red*) or depleted (*green*) relative to the obesity-related clinical markers collected in our study (KTwin) and in inflammatory bowel disease (IBD [13]) and/or type 2 diabetes (T2D [3]). Directionality of association in these common dysbioses was near-uniformly consistent, as indicated by box color

This pattern of microbial functional enrichments during inflammation has now been consistent across multiple studies regardless of their potential causality, including in other diseases like IBD (Fig. 6), suggesting a common signature of gut response to low-grade inflammation. Interestingly, several studies have examined whether inflammation can lead to obesity [45–47] and T2D [48–50] in mice, finding that inflammation drives the development of insulin resistance (potentially through the phosphorylation of insulin receptor 1 by a TNF-α mediated response [51]) and suggesting that particular intestinal microbial configurations can promote immune responses driving metabolic dysfunction [51]. The extent to which the gut microbiota causes obesity is an area of active research. Many mouse models, including *Lep*^*ob*^, consistently demonstrate an elevated Firmicutes:Bacteroidetes (F:B) ratio in obese animals [5, 31]. In contrast, in human cohorts, the relationship has been much less consistent. The relationship between obesity and the F:B ratio has been reported as increased [5, 8], decreased [34], and others have reported no relationship [21, 52], indicating there is still great variability in current studies. This may arise either from technical issues, like different sample or data handling protocols, or from biological reasons, like true variation between the various cohorts.

Many microbial genes, particularly housekeeping genes, are transcribed at a basal level, and thus their measured DNA and RNA levels are well-correlated. In contrast, other classes of genes such as vitamin and amino acid biosynthesis are much more dynamically regulated, so DNA and RNA levels are less correlated [53]. While this study measured only DNA abundance of genes, concurrent examination of the subclinical biomarker meta-transcriptome would be an informative extension of this work.

Finally, a unique dataset comprising shotgun metagenomes of MZ twins over time enabled us to find that although twins are more similar in their species composition, they often harbor different strains. This unexpected discrepancy between the microbial population structures of strains versus species in the human microbiome should be further explored, as it can be explained by a variety of very distinct ecological and molecular hypotheses. Hosts with similar genetic profiles may exert a modest but continuous selective pressure for the acquisition and maintenance of similar species in the gut, which could be tested by collecting data on the degree of shared early life and persistent environment versus genetics. Alternatively, initially identical strains acquired from a shared environment, possibly in early life, may be maintained but diverge through fixation of genetic drift and laterally transferred elements over time. Occasional strain differences within the same individual over time suggest a fast divergence rate; however, a larger study, with temporally dense sampling of both adults and infants, will be needed to address this question.

## Conclusions

To conclude, this study provides evidence of low-grade inflammation of the gut with increasing values of obesity- and T2D-related biomarkers. Compositional and functional microbial signatures indicate the presence of sub-clinical inflammation in adults increasingly at risk of these conditions, even before they are reflected by clinical markers. If these microbial shifts play a causal role in the onset of obesity or T2D, they may represent not only novel markers for early diagnosis, but also a target for preventative therapeutic intervention. Even if these shifts are not ultimately the primary causal agents behind their associated diseases, microbial dysbioses may still be manipulated to avert disease onset, and their specifics are likely to improve our mechanistic understanding of host-microbiota interaction and its role in disease prevention and treatment.

## Additional files

Additional file 1: Table S1. The clinical markers collected at each time a sample was taken. (XLSX 36 kb) Additional file 2: Figure S1. Distributions of selected clinical variables in our data (colorful) and as collected by the human microbiome project [22] (gray). (PDF 3586 kb) Additional file 3: Table S2. Prevalence and relative abundance of microbes and KEGG modules found in both this study and HMP [21]. (XLSX 31 kb) Additional file 4: Figure S2. The average microbial prevalence (**A**) and abundance (**B**) as compared between the Human Microbiome Project [21] and our data. Prevalence was defined as percent of samples with >0.001 relative abundance for each species, and average abundance was calculated only for samples passing that criterion. (PDF 3577 kb) Additional file 5: Figure S3. Hierarchical clustering of the (**A**) phylogenetic and (**B**) functional profiles of the samples. Profiles (rows) are colored by the cluster assignment (k = 15), and samples (columns) are colored by the twin variable. The medoid profile per cluster (written in black) was chosen as a representative and shown on Fig. 2. (PDF 3597 kb) Additional file 6: Table S3. All significant associations between the clinical markers and microbes and KEGG modules found in our data. (XLSX 35 kb) Additional file 7: Figure S4. Correlation between selected clinical variables in our data. (PDF 3598 kb) Additional file 8: Figure S5. Scatter plots of data collected for the Human Microbiome Project [22] for (**A**) blood pressure vs. *M. smithii* abundance and (**B**) BMI vs. *E. sireum*. In our data, both these associations have a threshold-like behavior, however this is not observed in the HMP data [21]. (PDF 3520 kb) Additional file 9: Figure S6. Scatter plots of selected associations of microbial (x-axis) and functional (y-axis) profiles. Each plot represents a single entry in the correlation matrix at Fig. 4, and the R^2 value of this correlation dictates the color of the matrix entry. (PDF 3643 kb) Additional file 10: Figure S7. The genomic contribution of the microbial-to-functional associations. (**A**) Each entry denotes the fraction of genomic reference sequences of a specific microbe (column) that has genes from a specific functional module (row). This matrix accounts for most of the significant correlations we find on Fig. 4. (**B**) Taxa co-occurrence matrix in our data, using Spearman correlation. (ZIP 6726 kb) Additional file 11: Figure S8. A correlation matrix comparing the microbial and functional profiles, similar to that show in Fig. 4, calculated for the Human Microbiome Project [21] data. (PDF 3729 kb) Additional file 12: Figure S9. Marker genes abundance profiles for the Human Microbiome Project data [21] of (**A**) *Akkermansia muciniphila* and (**B**) *Dialister invisus*, formatted as in Fig. 5b and c with differential markers colored in orange. (PDF 3628 kb) Additional file 13: Figure S10. Marker genes abundance profiles of (**A**) *Akkermansia muciniphila*; (**B**) *Roseburia intestinalis*; (**C**) *Bifidobacterium bifidum*; (**D**) *Faecalibacterium prausnitzii*, formatted as in Fig. 5b and c. (ZIP 6685 kb) Additional file 14: Figure S11. The strain similarity of present microbes in our data, estimated by the Bray-Curtis distance between self-samples over time (x-axis) and twin samples (y-axis). Species highlighted in bold are shown in detail on Fig. 5b and c and Additional file 13: Figure S10. (PDF 3544 kb)
