# Supplementary figures and images for: Sub-clinical detection of gut microbial biomarkers of obesity and type 2 diabetes
Source: Genome Med. 2016 Feb 17;8:17. doi: 10.1186/s13073-016-0271-6 (PMC4756455; doi:10.1186/s13073-016-0271-6)

# Supplementary Figure 1

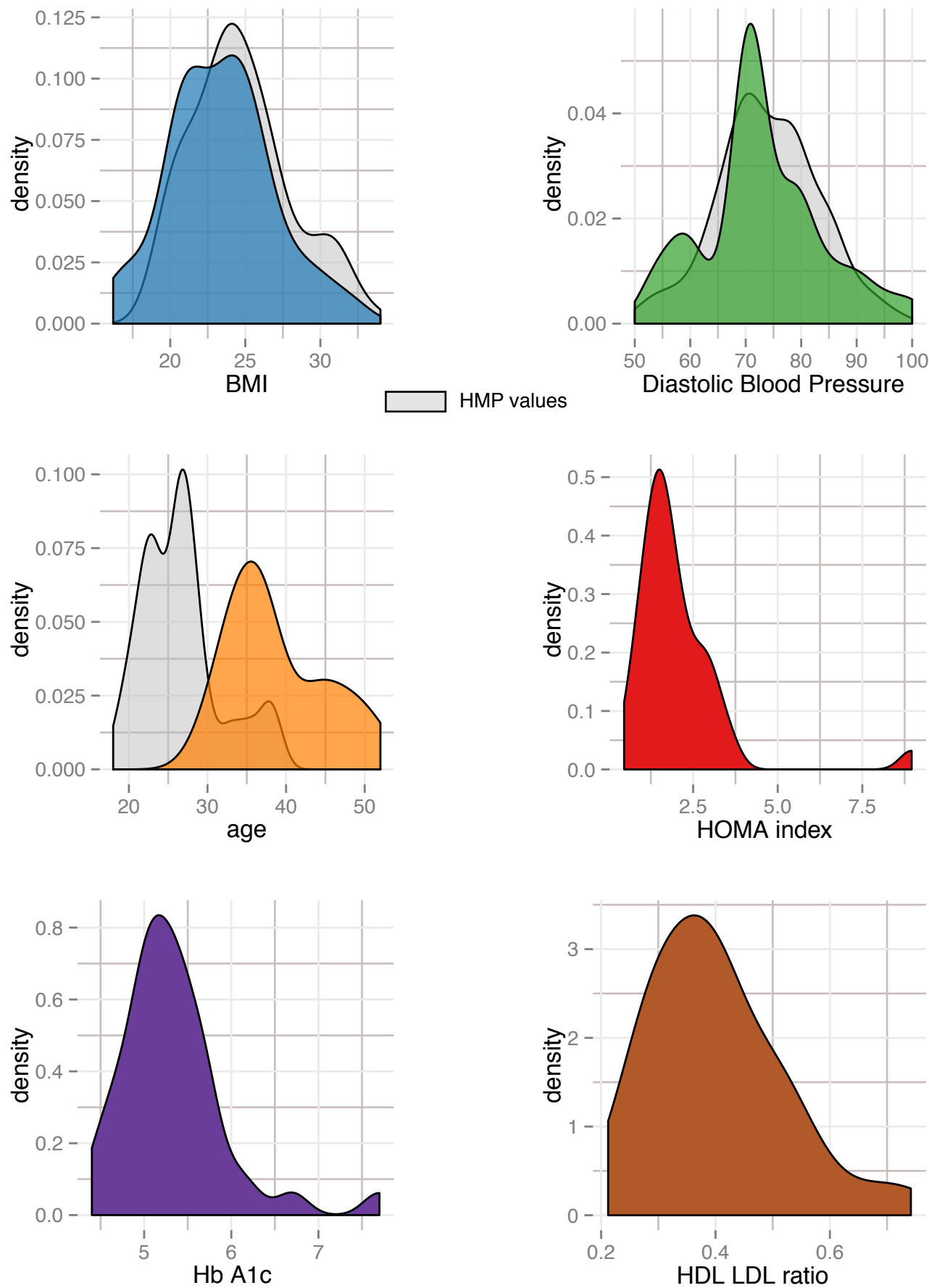

Supplement: Additional file 2: Figure S1. — Distributions of selected clinical variables in our data (colorful) and as collected by the human microbiome project [22] (gray). (PDF 3586 kb) [file 13073_2016_271_MOESM2_ESM.pdf]

# Supplementary Figure 3

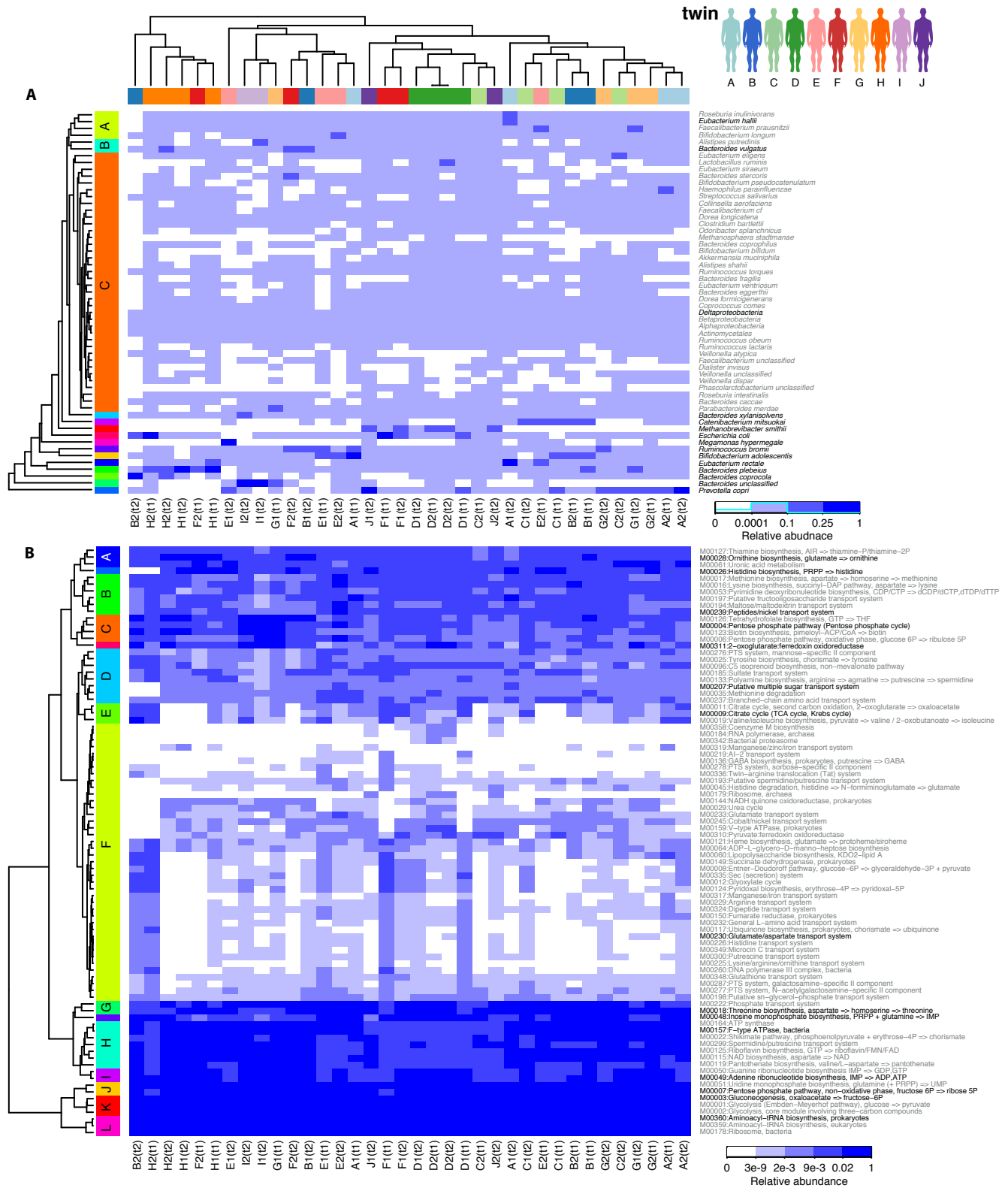

Supplement: Additional file 5: Figure S3. — Hierarchical clustering of the (A) phylogenetic and (B) functional profiles of the samples. Profiles (rows) are colored by the cluster assignment (k = 15), and samples (columns) are colored by the twin variable. The medoid profile per cluster (written in black) was chosen as a representative and shown on Fig. 2. (PDF 3597 kb) [file 13073_2016_271_MOESM5_ESM.pdf]

Supplementary Figure 4

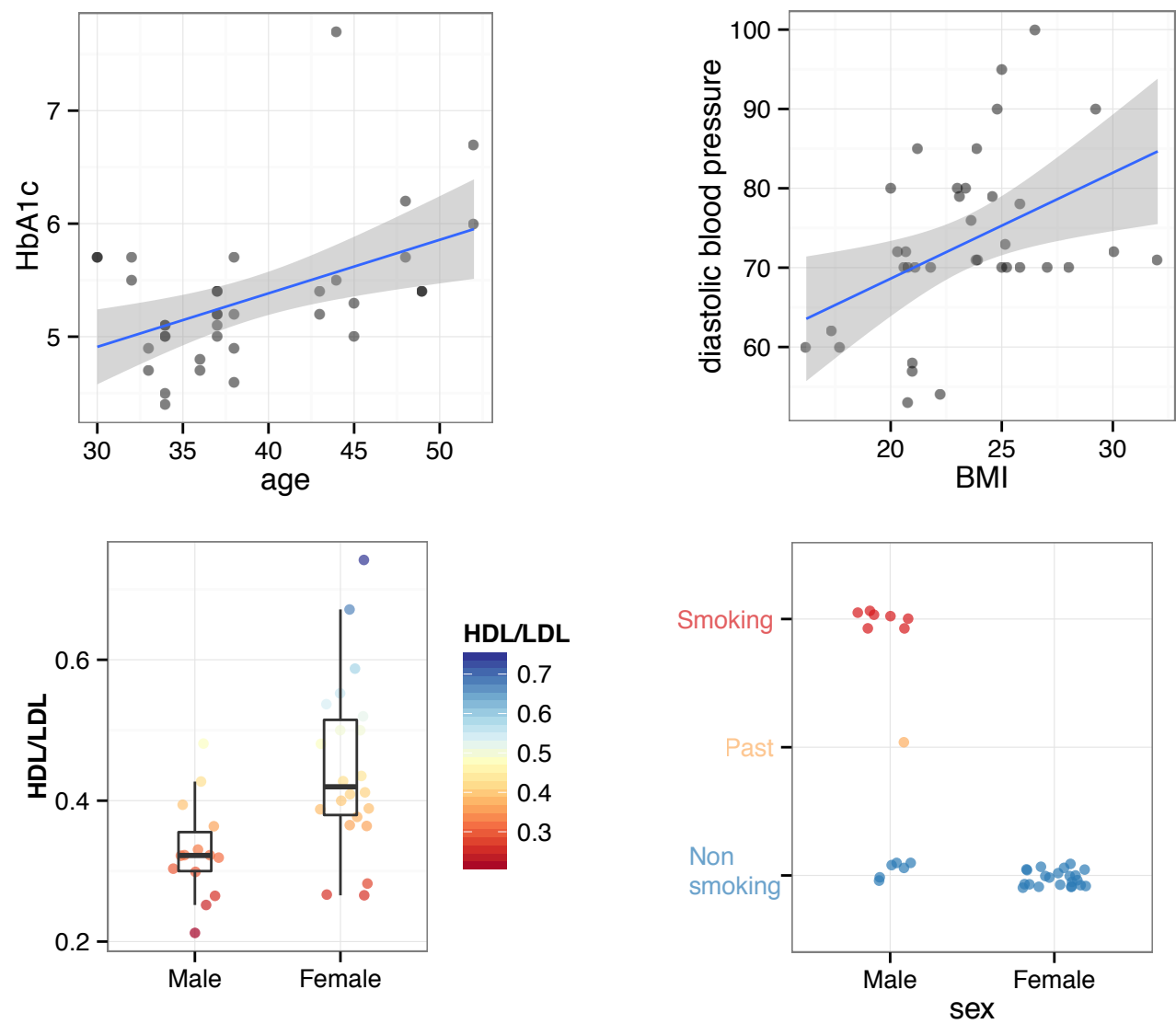

Supplement: Additional file 7: Figure S4. — Correlation between selected clinical variables in our data. (PDF 3598 kb) [file 13073_2016_271_MOESM7_ESM.pdf]

# Supplementary Figure 5

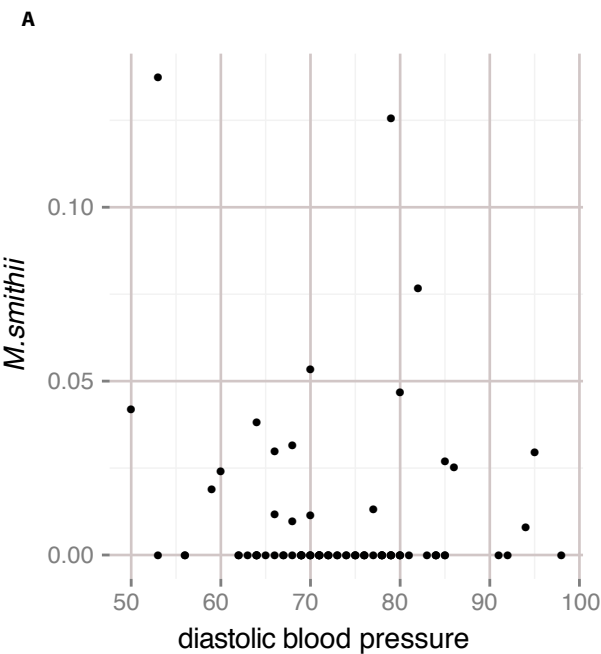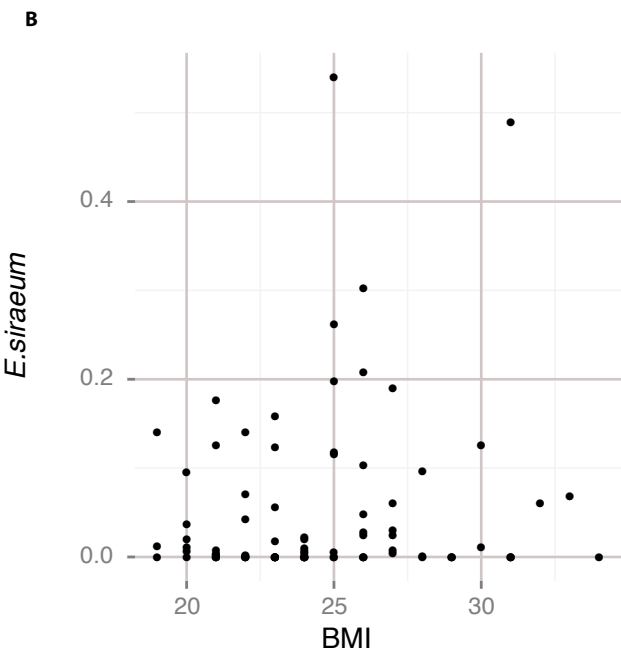

Supplement: Additional file 8: Figure S5. — Scatter plots of data collected for the Human Microbiome Project [22] for (A) blood pressure vs. M. smithii abundance and (B) BMI vs. E. sireum. In our data, both these associations have a threshold-like behavior, however this is not observed in the HMP data [21]. (PDF 3520 kb) [file 13073_2016_271_MOESM8_ESM.pdf]

# Supplementary Figure 6

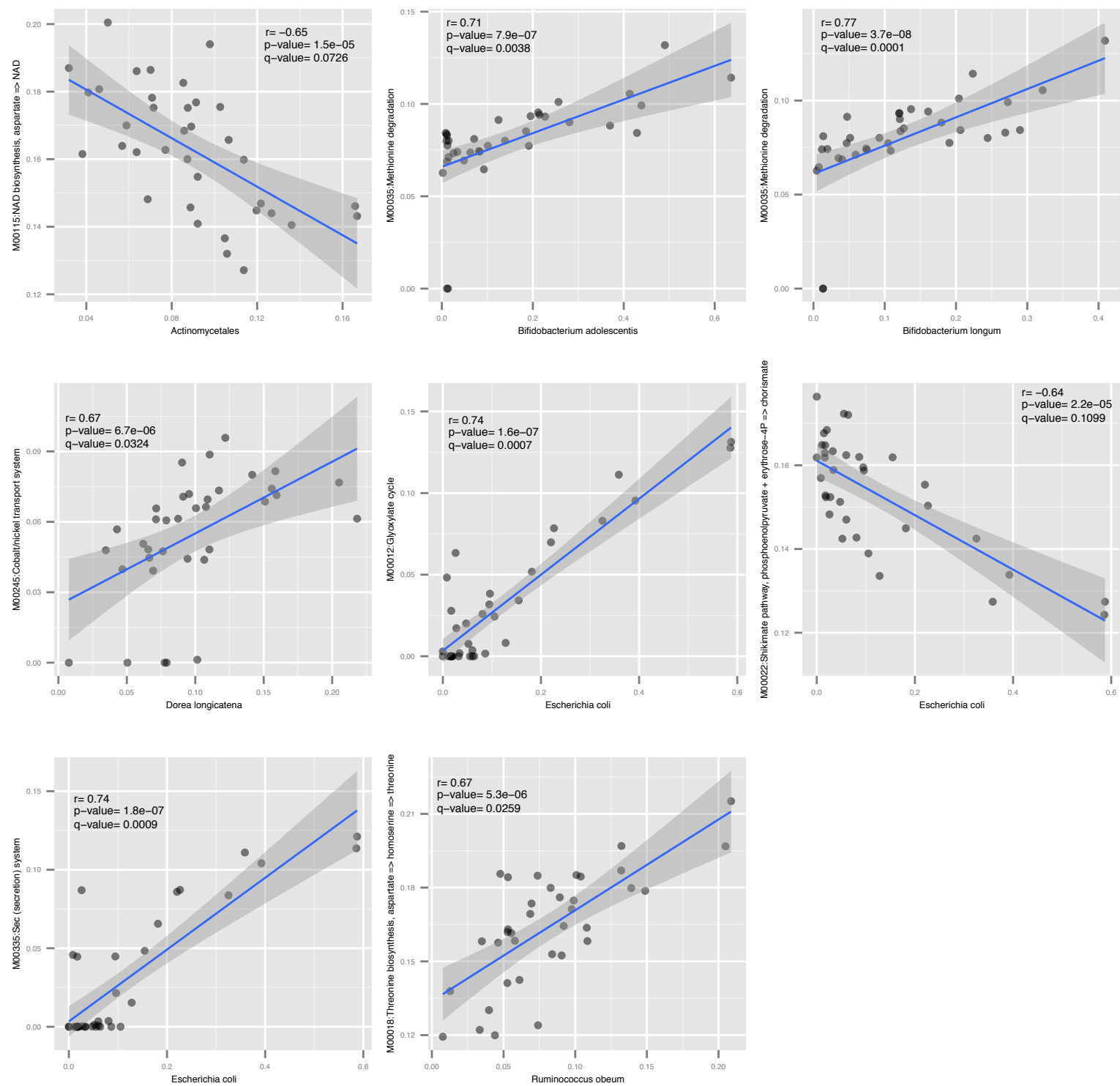

Supplement: Additional file 9: Figure S6. — Scatter plots of selected associations of microbial (x-axis) and functional (y-axis) profiles. Each plot represents a single entry in the correlation matrix at Fig. 4, and the R^2 value of this correlation dictates the color of the matrix entry. (PDF 3643 kb) [file 13073_2016_271_MOESM9_ESM.pdf]

Supplementary Figure 7A

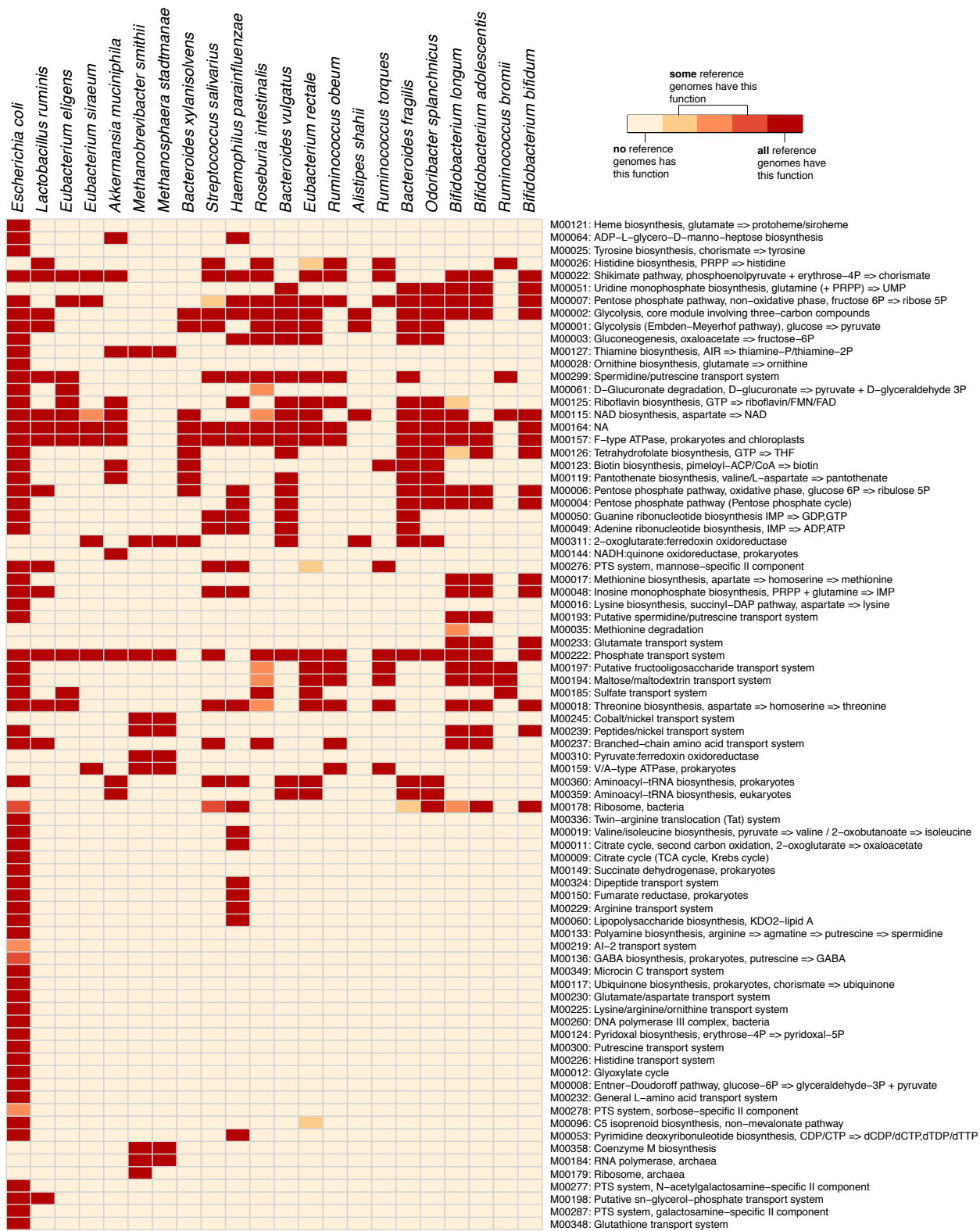

Supplement: Additional file 10: Figure S7. — The genomic contribution of the microbial-to-functional associations. (A) Each entry denotes the fraction of genomic reference sequences of a specific microbe (column) that has genes from a specific functional module (row). This matrix accounts for most of the significant correlations we find on Fig. 4. (B) Taxa co-occurrence matrix in our data, using Spearman correlation. (ZIP 6726 kb) [file 13073_2016_271_MOESM10_ESM.zip › Supp Figures 7.pdf]

Supplementary Figure 7B

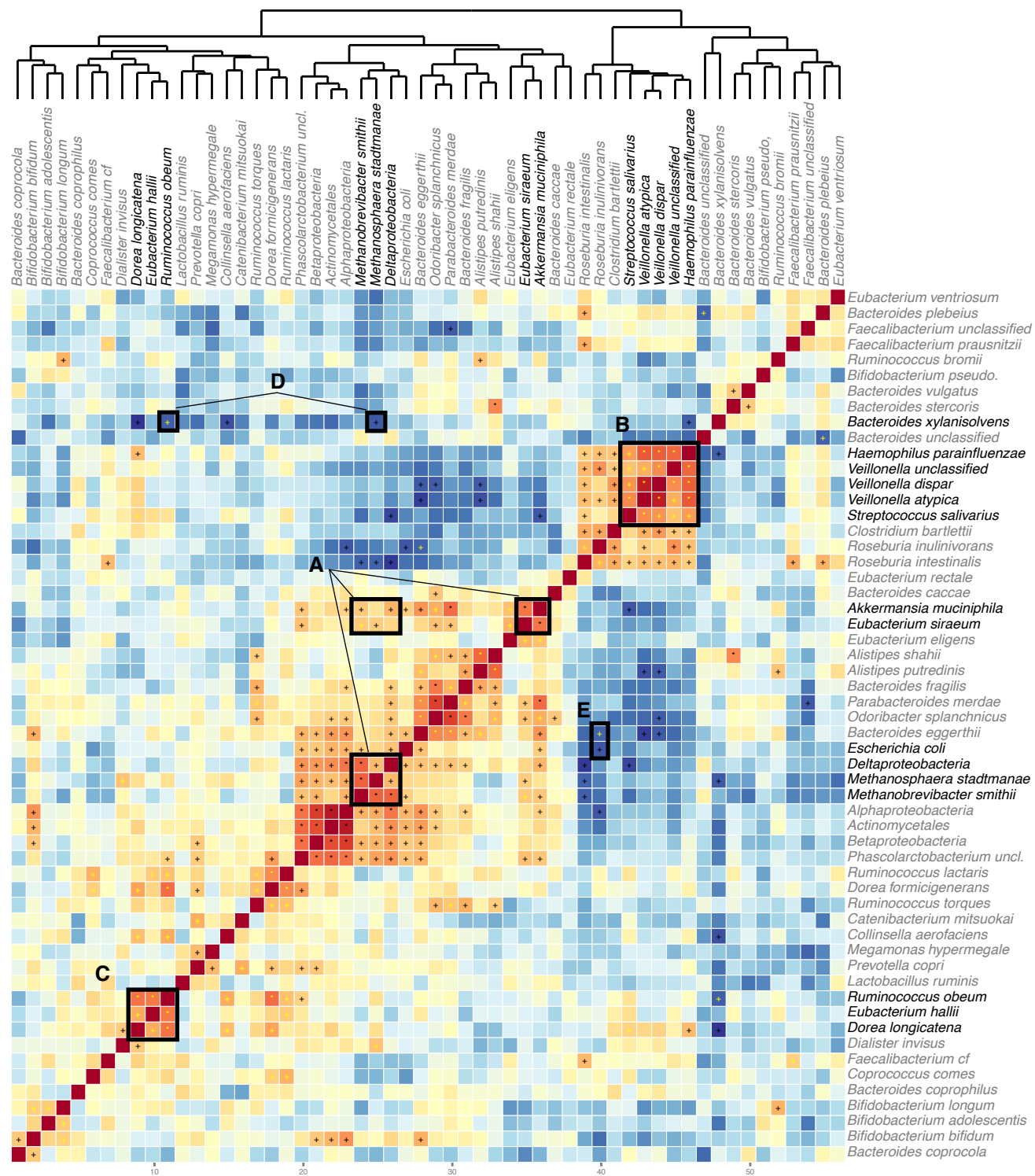

Supplement: Additional file 10: Figure S7. — The genomic contribution of the microbial-to-functional associations. (A) Each entry denotes the fraction of genomic reference sequences of a specific microbe (column) that has genes from a specific functional module (row). This matrix accounts for most of the significant correlations we find on Fig. 4. (B) Taxa co-occurrence matrix in our data, using Spearman correlation. (ZIP 6726 kb) [file 13073_2016_271_MOESM10_ESM.zip › Supp Figures 8.pdf]

## Supplementary Figure 8

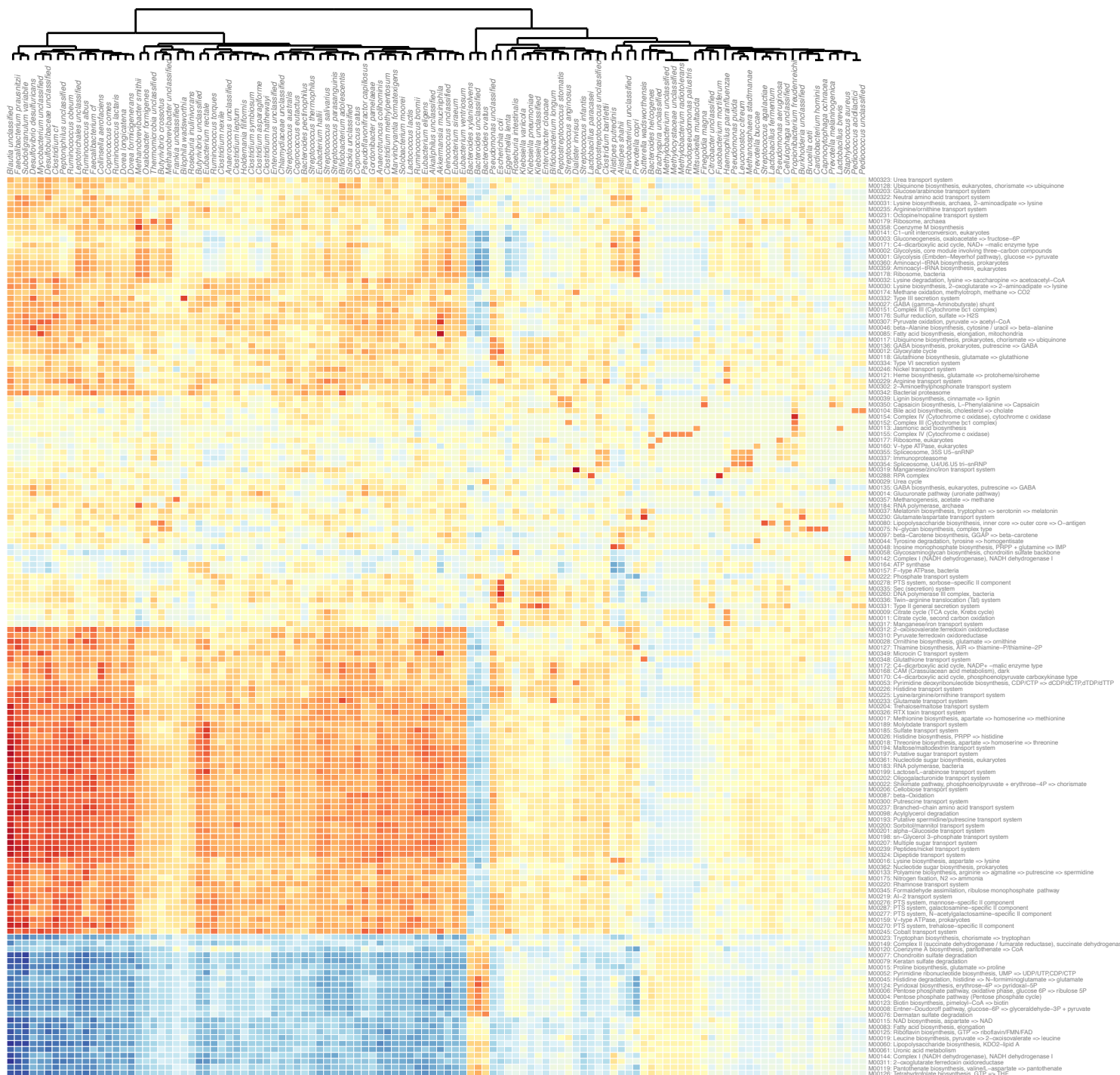

Supplement: Additional file 11: Figure S8. — A correlation matrix comparing the microbial and functional profiles, similar to that show in Fig. 4, calculated for the Human Microbiome Project [21] data. (PDF 3729 kb) [file 13073_2016_271_MOESM11_ESM.pdf]

# Supplementary Figure 9

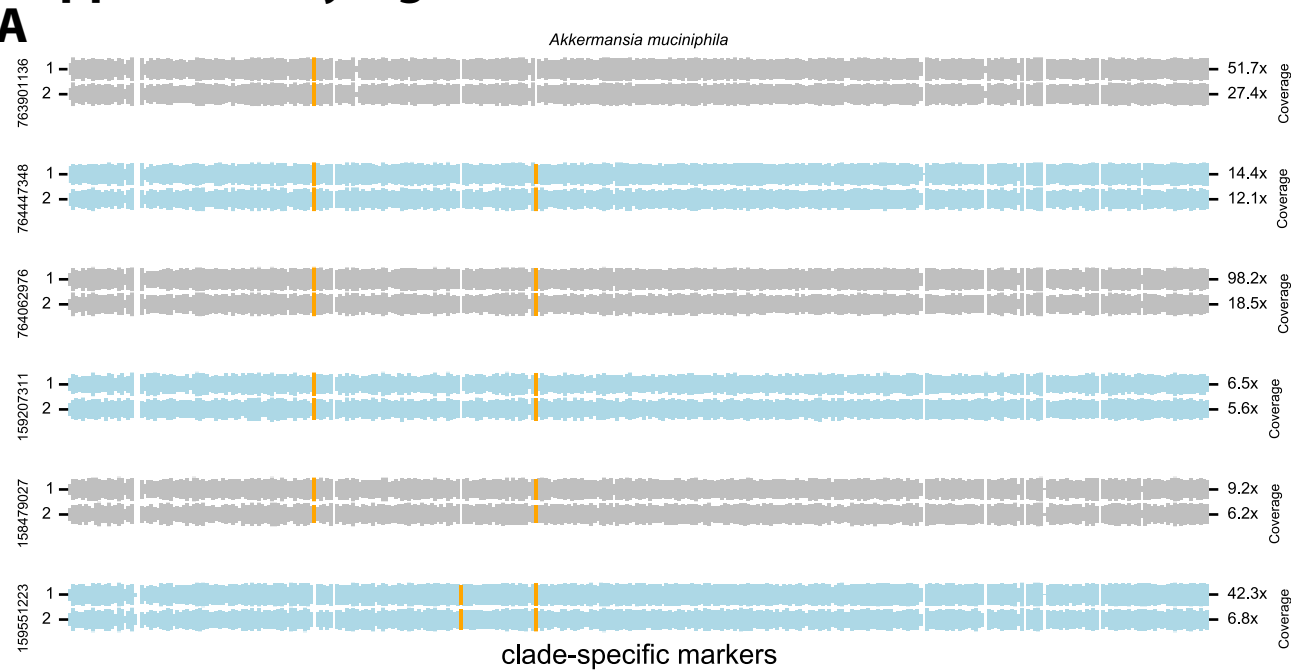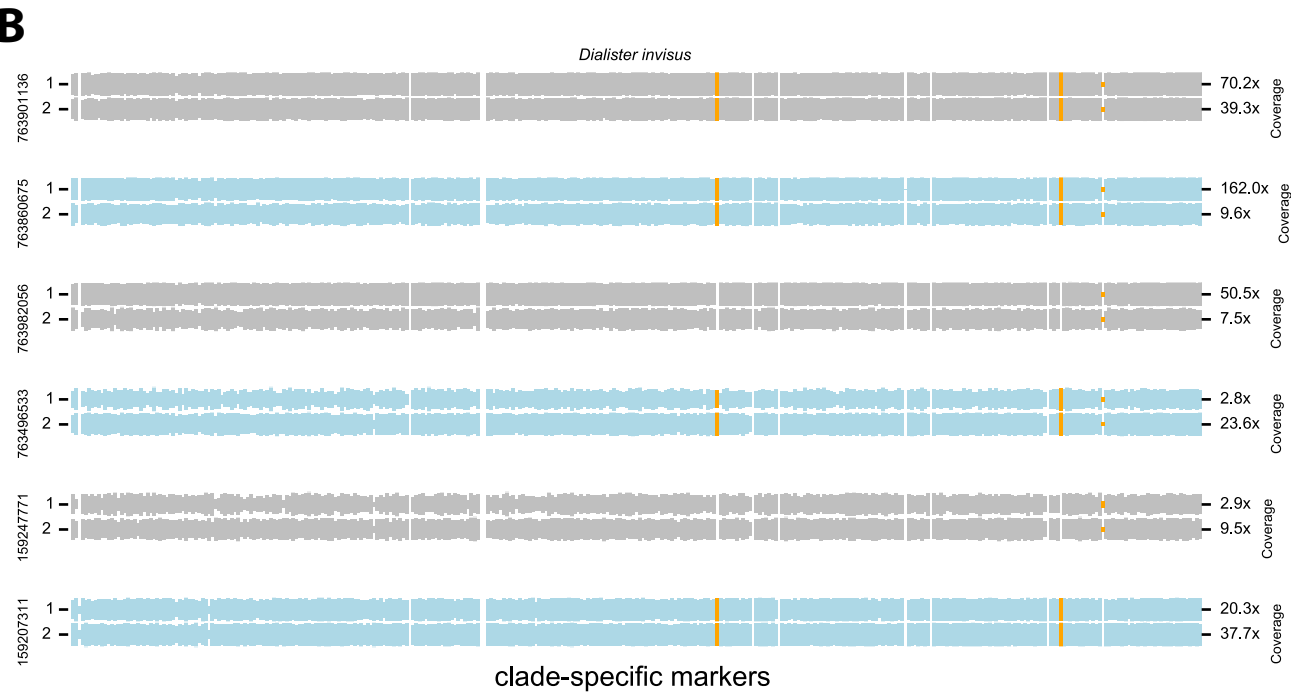

Supplement: Additional file 12: Figure S9. — Marker genes abundance profiles for the Human Microbiome Project data [21] of (A) Akkermansia muciniphila and (B) Dialister invisus, formatted as in Fig. 5b and c with differential markers colored in orange. (PDF 3628 kb) [file 13073_2016_271_MOESM12_ESM.pdf]

# Supplementary Figure 10A-C

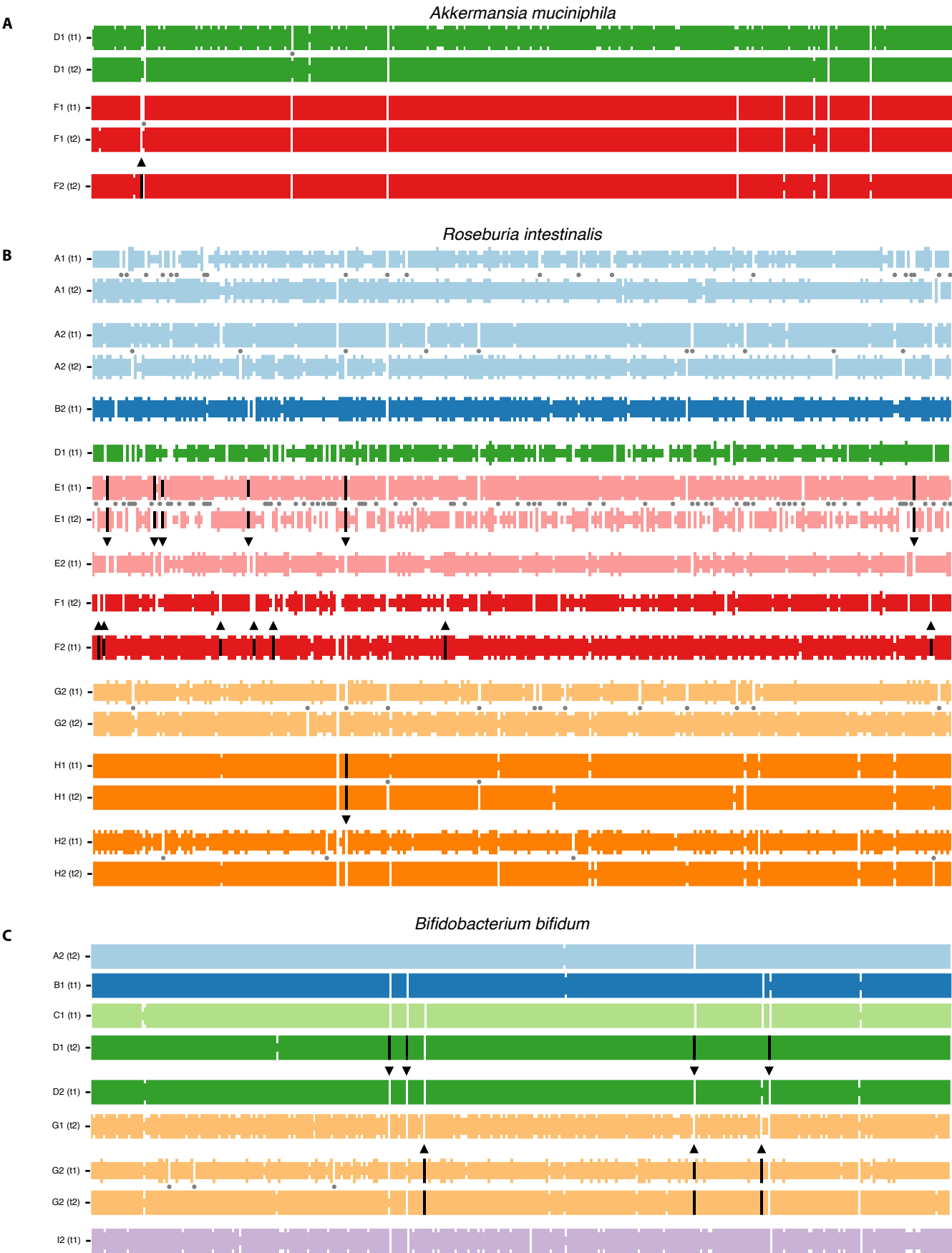

Supplement: Additional file 13: Figure S10. — Marker genes abundance profiles of (A) Akkermansia muciniphila; (B) Roseburia intestinalis; (C) Bifidobacterium bifidum; (D) Faecalibacterium prausnitzii, formatted as in Fig. 5b and c. (ZIP 6685 kb) [file 13073_2016_271_MOESM13_ESM.zip › Supp Figures 11.pdf]

Supplementary Figure 10D

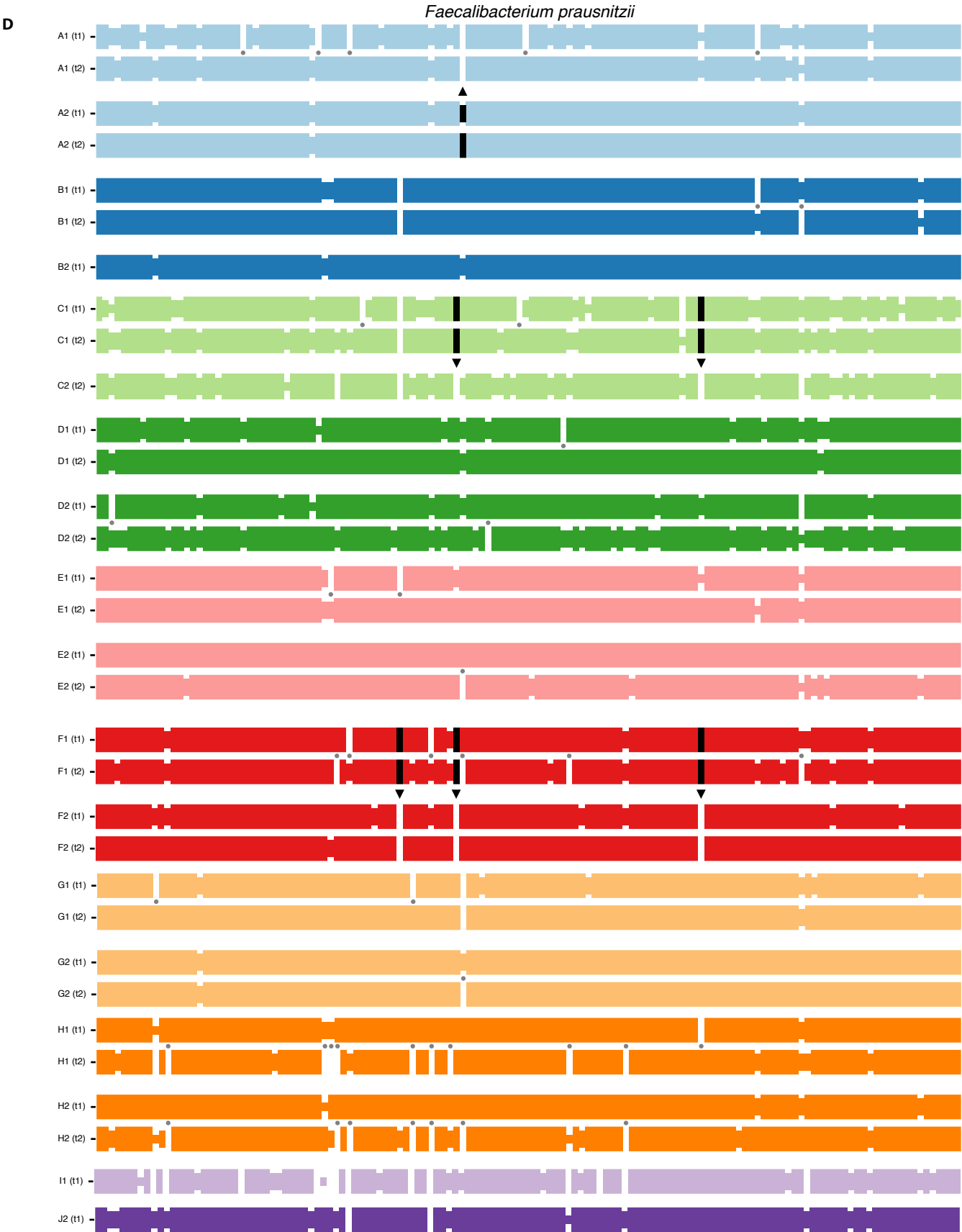

Supplement: Additional file 13: Figure S10. — Marker genes abundance profiles of (A) Akkermansia muciniphila; (B) Roseburia intestinalis; (C) Bifidobacterium bifidum; (D) Faecalibacterium prausnitzii, formatted as in Fig. 5b and c. (ZIP 6685 kb) [file 13073_2016_271_MOESM13_ESM.zip › Supp Figures 12.pdf]

# Supplementary Figure 11

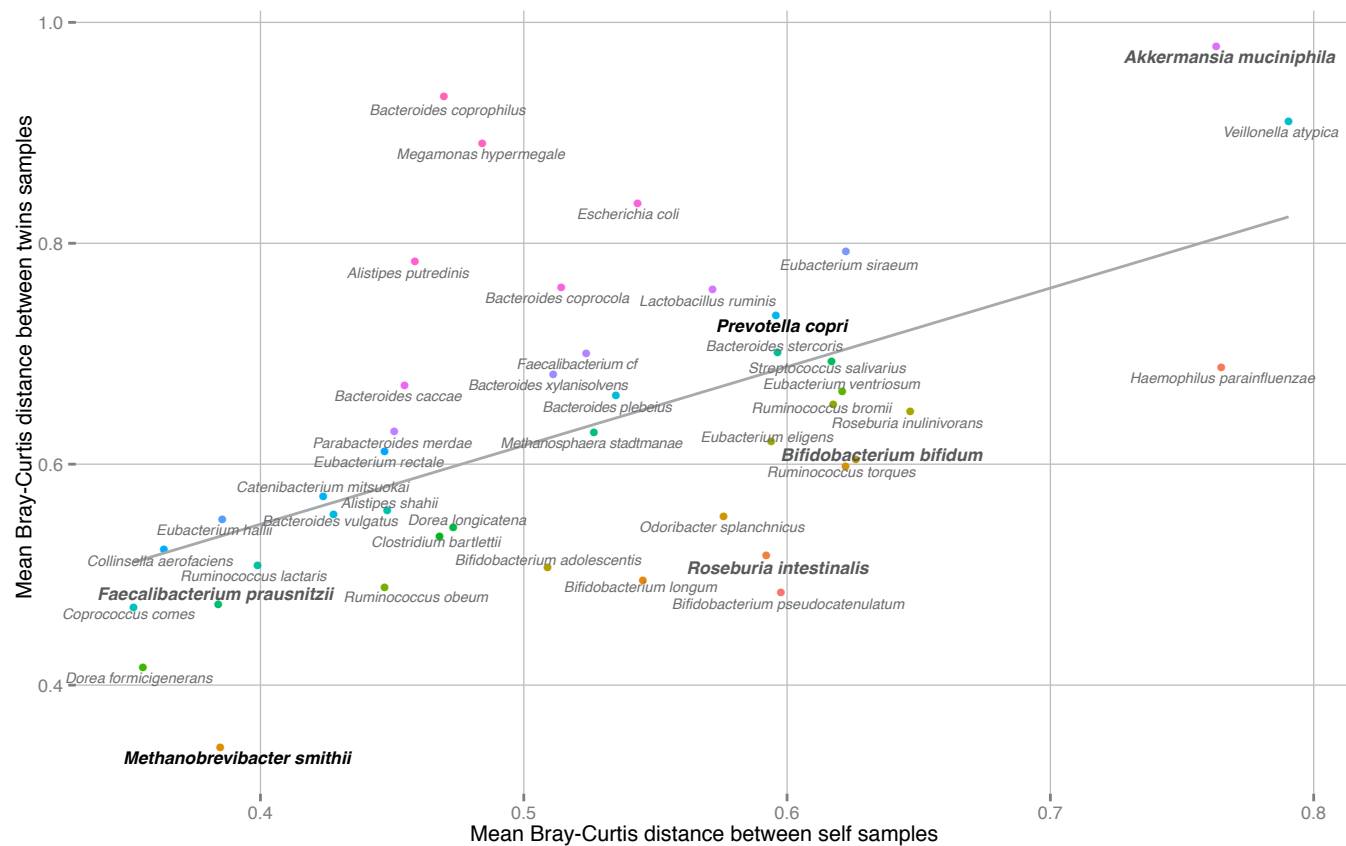

Supplement: Additional file 14: Figure S11. — The strain similarity of present microbes in our data, estimated by the Bray-Curtis distance between self-samples over time (x-axis) and twin samples (y-axis). Species highlighted in bold are shown in detail on Fig. 5b and c and Additional file 13: Figure S10. (PDF 3544 kb) [file 13073_2016_271_MOESM14_ESM.pdf]
