# Supplementary material for: Sub-clinical detection of gut microbial biomarkers of obesity and type 2 diabetes
Source: Genome Med. 2016 Feb 17;8:17. doi: 10.1186/s13073-016-0271-6 (PMC4756455; doi:10.1186/s13073-016-0271-6)

Supplementary Figure 2

A

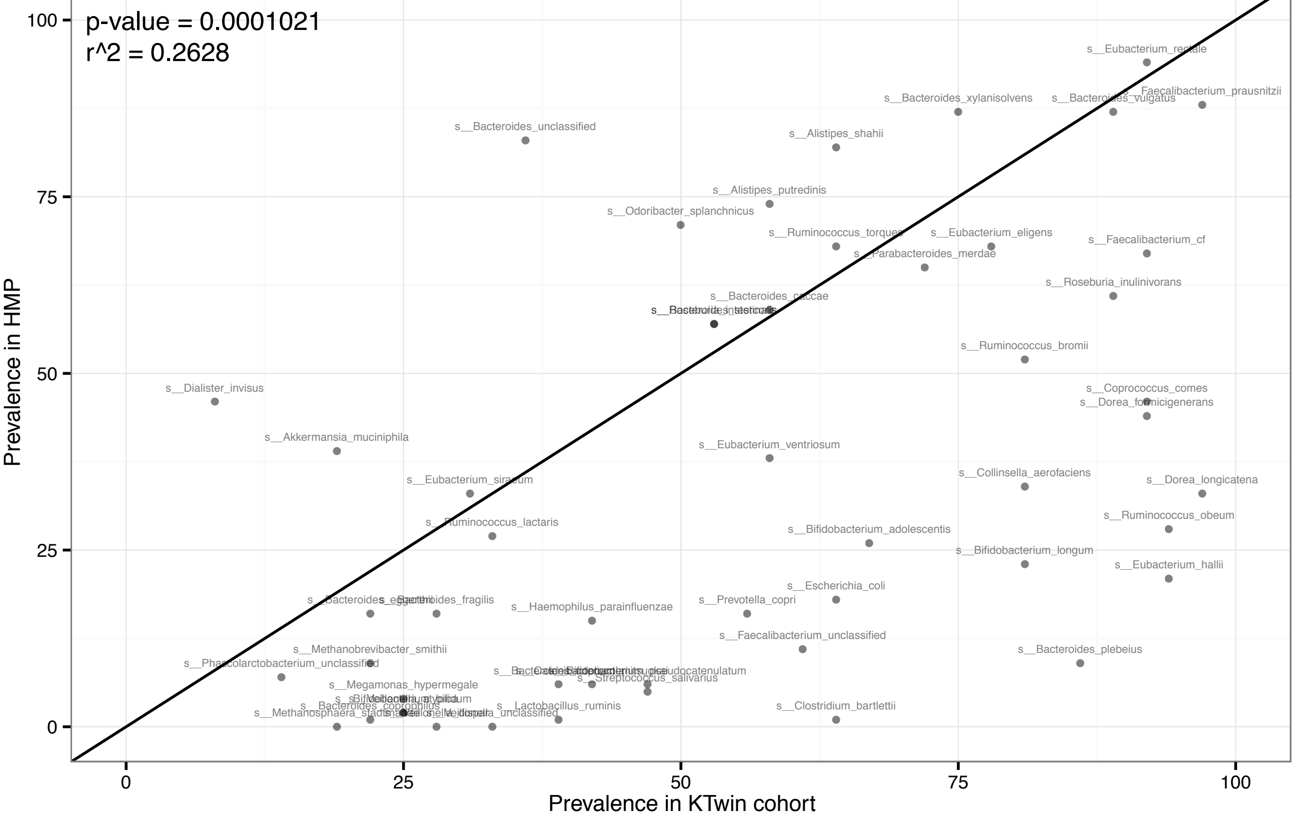

B

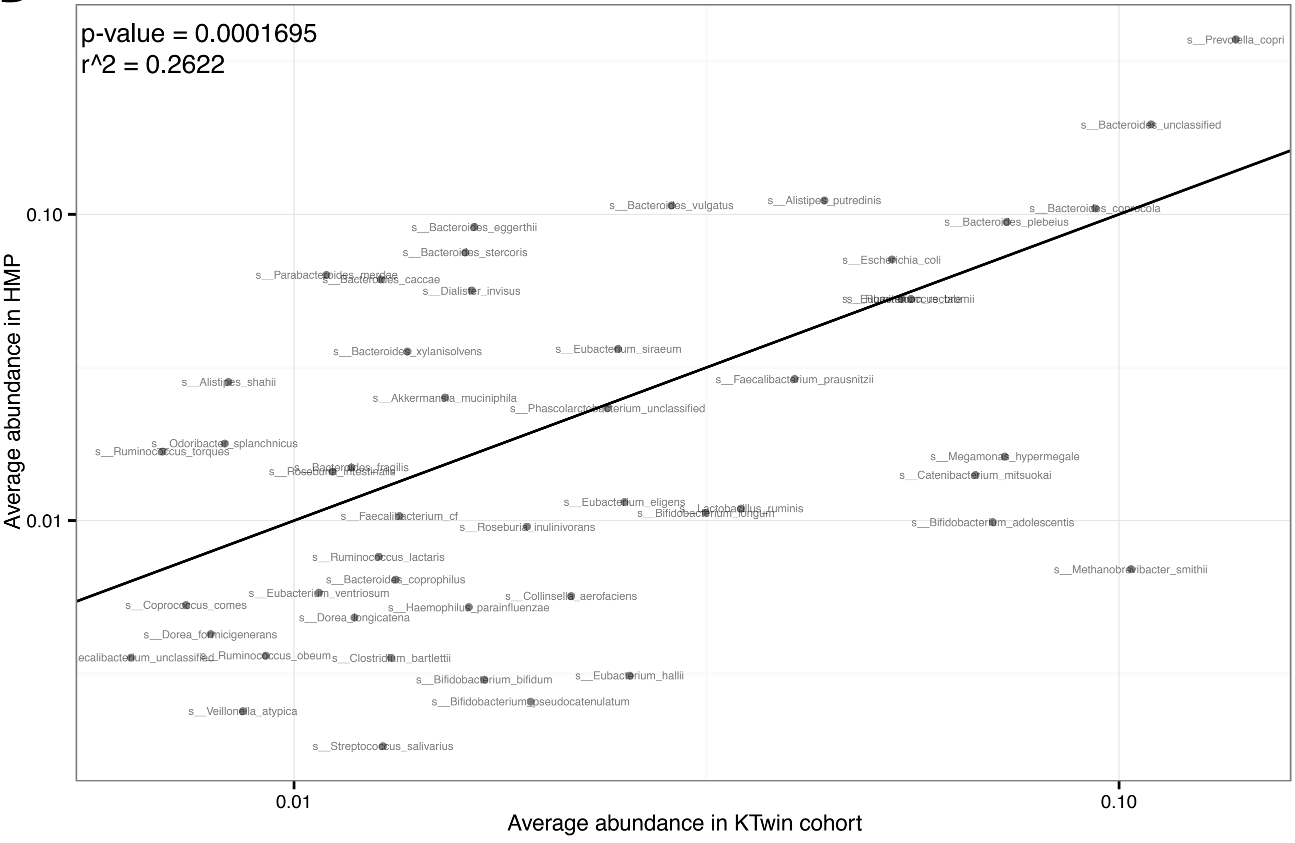

Supplement: Additional file 4: Figure S2. — The average microbial prevalence (A) and abundance (B) as compared between the Human Microbiome Project [21] and our data. Prevalence was defined as percent of samples with >0.001 relative abundance for each species, and average abundance was calculated only for samples passing that criterion. (PDF 3577 kb) [file 13073_2016_271_MOESM4_ESM.pdf]
